# A simple and efficient method to visualize and quantify the efficiency of chromosomal mutations from genome editing

**DOI:** 10.1038/srep35488

**Published:** 2016-10-17

**Authors:** Liezhen Fu, Luan Wen, Nga Luu, Yun-Bo Shi

**Affiliations:** 1 Section on Molecular Morphogenesis, Eunice Kennedy Shriver National Institute of Child Health and Human Development (NICHD), National Institutes of Health (NIH), 18 Library Dr., Bethesda, Maryland, 20892, United States.

## Abstract

Genome editing with designer nucleases such as TALEN and CRISPR/Cas enzymes has broad applications. Delivery of these designer nucleases into organisms induces various genetic mutations including deletions, insertions and nucleotide substitutions. Characterizing those mutations is critical for evaluating the efficacy and specificity of targeted genome editing. While a number of methods have been developed to identify the mutations, none other than sequencing allows the identification of the most desired mutations, i.e., out-of-frame insertions/deletions that disrupt genes. Here we report a simple and efficient method to visualize and quantify the efficiency of genomic mutations induced by genome-editing. Our approach is based on the expression of a two-color fusion protein in a vector that allows the insertion of the edited region in the genome in between the two color moieties. We show that our approach not only easily identifies developing animals with desired mutations but also efficiently quantifies the mutation rate *in vivo*. Furthermore, by using LacZα and GFP as the color moieties, our approach can even eliminate the need for a fluorescent microscope, allowing the analysis with simple bright field visualization. Such an approach will greatly simplify the screen for effective genome-editing enzymes and identify the desired mutant cells/animals.

With the recent advancement in designer nucleases, especially the TALEN and CRISPR/Cas enzymes^1^,^2^,^3^, genome-editing is rapidly finding applications in diverse biological and biomedical fields^4^,^5^,^6^,^7^,^8^. These various genome-editing tools allow easy introduction of mutations from base substitutions to insertions/deletions in cells and live organisms. As a single designer nuclease often introduces diverse mutations on the target gene, it is essential to identify the desired mutations, often out-of-frame insertions/deletions that disrupt the target gene. This is because in-frame mutations, including base substitutions and deletions/insertions, may produce proteins that retain some or all of the function of the wild type protein. A number of methods have been developed in recent years to facilitate the identification of mutations caused by designer nucleases. The enzyme mismatch cleavage assays (MCAs) such as Surveyor^9^ or T7 endonuclease I (T7E1)^10^ mismatch cleavage assay are widely adapted to quantify mutation rates as a primary evaluation of targeted mutagenesis by designer nucleases. Other methods, such as High Resolution Melt Assay (HRMA)^11^, droplet digital PCR (ddPCR)^12^, and LacZ recovery/disruption assay^13^, have also been applied with varied benefits and disadvantages. These methods allow relatively easy identification of cells or animals with mutations in the targeted regions and in some case, an estimate of the mutation efficiency caused by the genome-editing enzymes. Since often only the out-of-frame mutations ensure the disruption of the gene of interest, sequencing of the targeted region is required to determine if such mutations are present.

We have developed a very simple and efficient method to visualize and quantify the efficiency of the mutations induced by genome-editing. Our approach is to make use of the ability of protein moieties such as GFP retain its function independently even when present in a fusion protein. Thus, we make a construct to express a two-color fusion protein, such as mCherry-GFP with an in-frame cloning linker containing several restriction enzyme recognition sites. This allows us to easily insert gene-edited region in between the two color moieties. Out-of-frame deletions or insertions within the gene-edited region will disable the expression of the second color moiety, e.g., GFP, and thus can be easily detected based on a simple visualization of the bacterial colonies under a microscope. By using *Xenopus tropicalis* as a model system, we show that this approach allows easy identification and efficient quantification of the desired (out-of-frame) mutations in developing animals. We envision that such an approach will be highly valuable for screening effective genome-editing enzymes and mutant cells/organisms for functional studies/applications *in vitro* and in animals.

## Materials and Methods

### Animal care and embryo microinjection

*Xenopus tropicalis* adult frogs were purchased from NASCO (Fort Atkinson, WI, USA). All animal care and treatment were done as approved by Animal Use and Care Committee of Eunice Kennedy Shriver National Institute of Child Health and Human Development (NICHD), U.S. National Institutes of Health (NIH). The methods were carried out in accordance with relevant guidelines for the use of Xenopus tropicalis as a vertebrate model. The embryos for microinjection were prepared essentially as previously described by using mature adult *Xenopus tropicalis* frogs^14^. Briefly, a few females and a male were primed with 20 U of human chorionic gonadotropin (hCG; Novarel; Ferring Pharmaceuticals Inc. Parsippany, NJ, USA) 1 day before the experiment. The injected frogs were boosted with 200 U of hCG on the second day. Just before the females started to lay eggs, the male was sacrificed to obtain testes. One testis was smashed to prepare a sperm suspension in 300 μl 1 × MMR. For *in vitro* fertilization, freshly squeezed eggs (3–5 ml total volume equivalent) from an hCG-injected female were mixed with 100 μl the sperm suspension for about 2 min. The sperms in the mixture were then activated by diluting the mixture with 900 μl H_2_O. The fertilized eggs were dejellied in 3% cysteine in 0.1 × MMR (pH 8.0). After washing with 0.1 × MMR several times, the fertilized eggs were placed on an agar-coated plate. For TALEN mRNA injection, equal amounts of the TALEN-L (left) and -R (right) arm mRNAs were mixed and injected into the fertilized egg at 400 pg for each mRNA/egg.

### Construction of plasmids

The plasmid expressing a fusion protein of mCherry and GFP (pmCherry-GFP) was constructed from pUC19 (Invitrogen, Waltham, MA, USA) by inserting the mCherry coding sequence between the SalI and HindIII sites of the multi-cloning sites (MCS) of the pUC19 to allow in-frame expression of mCherry and the LacZα gene in pUC19, and then replacing the LacZα coding region with super folding green fluorescent gene (sfGFP, GenBank # HQ873313). Briefly, mCherry coding sequences were PCR amplified from plasmid Nanog-2A-mCherry (a gift from Dr. Rudolf Jaenisch (Addgene plasmid # 59995)^15^ with primers 5′-cggcgcagaGTCGACttgtacagctcgtccatgcc-3′ (*Sal*I site capitalized) and 5′-gattacgccAAGCTTgatggtgagcaagggcgaggaggata-3′ (*Hind*III site capitalized). The amplified DNA was double digested with *Sal*I and *Hind*III followed by gel-purification and inserted into pUC19 vector linearized with the same two restriction enzymes. The resulting construct was verified by sequencing. The construct was then digested with NdeI and end filled with *E. coli* DNA Polymerase I Klenow fragment to produce linearized DNA with blunt ends. The DNA was re-digested with EcoRI to remove the LacZα coding region. The vector portion, lacking the LacZα coding region, was gel-purified for subsequent insertion of the sfGFP coding sequence. The sfGFP coding sequence was PCR amplified from a synthetic sfGFP gene (Eurofin Genomics, Huntsville, AL, USA) with primers 5′-ccgagctc*gaattc*aactggccgtcgttttacCGGTACCATGCGTAAAGGC-3′ (*EcoR*I in italic letters) and 5′-TCATCATTTGTACAGTTCATCCAT-3′ followed by EcoRI digestion and gel-purification. The sfGFP fragment was then ligated into the gel purified vector containing the mCherry through the EcoRI site at the 5′-end of the sfGFP fragment and blunt end at the 3′-end to produce pmCherry-GFP.

To generate the construct to express fusion protein of LacZα-GFP, the mCherry coding sequence in pmCherry-GFP was replaced with the LacZα coding sequence. Briefly, the LacZα coding sequence was PCR amplified from pUC19 with primers 5′-ttacgccaagcttgctagcggtagtgttacaacg-3′ (*Hind*III site underlined) and 5′-ctctagagtcgacgatgcggcatcagagcagattg-3′ (*Sal*I site underlined). The PCR product and pmCherry-GFP were digested with HindIII and SalI, which removed the mCherry sequence from the pmCherry-GFP vector. The resulting linearized vector and the LacZα fragment was ligated together to produce pLacZα-GFP, which expresses LacZα-GFP fusion protein.

All PCR reactions were carried out with high fidelity PrimeSTAR GXL DNA Polymerase (Clontech, Mountain View, CA, USA).

### TALEN assembly and TALEN mRNA preparation

The TALEN pair targeting *Xenopus tropicalis* TRα DNA binding domain (left and right arms, or TRα-L and TRα-R) was described previously^16^. A TALEN pair targeting *Xenopus tropicalis* Sox3 were custom-designed and assembled by Cellectis Bioresearch, Inc. (Cambridge, MA, USA) to target the region around the start codon of the gene. The Sox3 TALEN left arm (Sox3-L) recognizes the sequence 5′-TCCTCCACCTGCAGCTC-3′ on the sense strand and the Sox3 TALEN right arm (Sox3-R) recognizes the sequence 5′- TTGAGGTCTGTGTCCAA-3′ on the antisense strand.

To generate the TALEN mRNAs *in vitro*, the individual TALEN plasmid was linearized with NotI (for TRα-L and TRα-R) or PacI (for Sox3-L and Sox3-R). Capped mRNA was produced by using the linearized DNA and the Ambion (Grand Island, NY, USA) *in vitro* transcription kit. After removing the DNA template by DNaseI digestion, capped mRNA was purified by RNAeasy kit (Qiagen, Valencia, CA, USA).

### PCR amplification and cloning of genomic DNA for visualization detection

For detection and screening of TALEN-induced out-of-frame mutations, genomic DNA was isolated from TALEN mRNA-injected embryos 3 to 5 days after fertilization and subjected to first round PCR amplification by using primers listed in Table 1 for 15 cycles. The PCR products were diluted at 1:1000 and subjected to the second round PCR amplification with nested primers (Table 1) for 25 to 30 cycles. The PCR products were analyzed by agarose-gel electrophoresis to check specificity, followed by purification by using the PCR Purification Kit (Qiagen, Valencia, CA, USA). The products were subjected to BamHI and EcoRI double digestion at 37 °C for at least 2 hours and then treated with calf intestinal alkaline phosphatase (CIP) (NEB, MA, USA) for 10 min. The dephosphorylated PCR products bearing a BamHI and an EcoRI ends were gel-purified with the Gel Extraction Kit (Qiagen, Valencia, CA, USA) and ligated into pmCherry-GFP or pLacZα-GFP predigested with BamHI and EcoRI. The dephosphorylation of the PCR products prevented the cloning of more than one insert into a vector.

### Transformation, photography, and out-of-frame mutation rate analysis

The recombinant DNA generated above was transformed into *E. coli* TOP10 competent cells (Invitrogen, Waltham, MA, USA), activated at 37 °C for 30 min with shaking, and spread on agar plates containing ampicillin and IPTG (for pmCherry-GFP) or IPTG/Xgal (for pLacZα-GFP). After incubation at 37 °C overnight, the plates were observed under a fluorescent dissection microscope (Model MZ10F, Leica, Buffalo Grove, IL, USA) and photographed with a MicroPublisher 5.0 digital camera attached to the microscope (QImagining, Surrey, BC, Canada) under bright field or fluorescent conditions. The imagines were processed with Adobe PhotoShop (San Jose, CA, USA). To determine the out-of-frame mutation rate, the transformed bacteria were seeded at a density of about 40 to 300 colonies per plate with at least 3 duplicates for each strain of bacterial transformants and visualized under fluorescent conditions to count GFP-negative colonies and total colonies of mCherry-positive (for pmCherry-GFP based assay) or blue/greenish blue colonies (for pLacZα-GFP based assay) on the same plate, respectively. The out-of-frame mutation rates were calculated by dividing the numbers of GFP-negative colonies by the total number of the mCherry-positive colonies (for pmCherry-GFP based assay) or blue/greenish blue colonies (for pLacZα-GFP based assay) on the same plates, respectively, and presented as mean with standard error (Mean ± S.E., Table 2).

### DNA sequencing

Individual bacterial colonies were picked up from plates under the bright field or fluorescent dissection microscope and grown in LB medium supplemented with ampicillin overnight at 37 °C with shaking at 220 rpm. Plasmid DNA was purified from the cultures with QIAprep Spin Miniprep Kit (Qiagen, Valencia, CA, USA) and sequenced (Eurofin Genomics, Huntsville, AL, USA). DNA sequence alignment was performed by using MegAlign of DNASTAR (Madison, WI, USA).

## Results and Discussions

### A fusion protein of mCherry and GFP with an in-frame linker retains both mCherry and GFP fluorescence when expressed in bacteria

**Figure 1:**
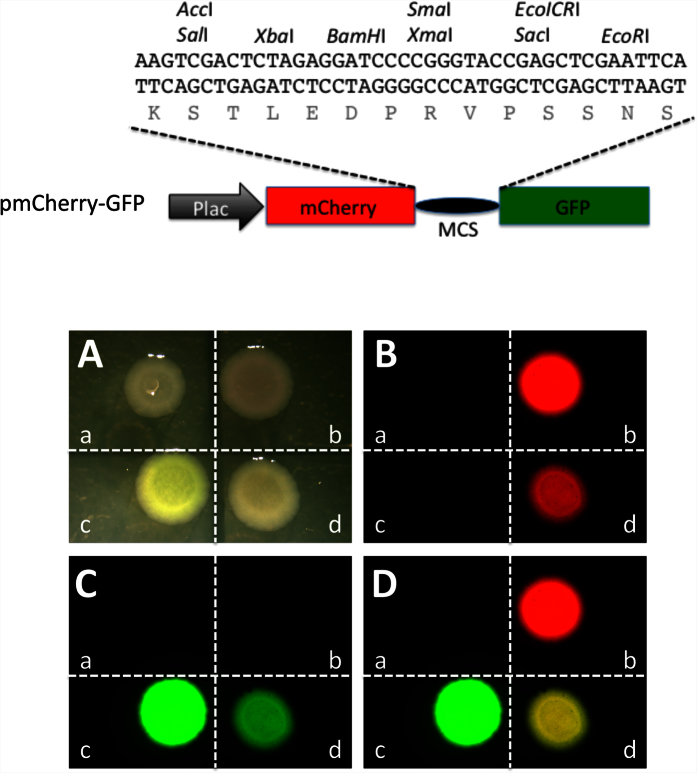
A construct containing mCherry and GFP coding regions separated by an in frame linker sequence allows the expression of a fusion protein in which the mCherry and GFP moieties fluoresce independently in bacteria. Top panel: Schematic representation of a plasmid construct for in-frame expression of mCherry and GFP (pmCherry-GFP). A sequence derived from pUC19 that contains multi-cloning sites (MCS) was inserted in between the mCherry and GFP coding regions under the control of the promoter of the bacterial lac operon (Plac). The inserted MCS sequence allows in frame translation of both mCherry and GFP separated by a linker peptide encoded by the MCS as shown. Bottom panel: When transfected into bacteria, pmCherry-GFP allows the expression of a fusion protein with both the mCherry and GFP moieties functioning independently. Bacteria were transformed with empty vector pUC19 (a), constructs expressing only mCherry (b) or GFP (c), or pmCherry-GFP (d), respectively. The transformed bacteria were plated and imaged in the bright field (**A**), or under a fluorescent microscope for red fluorescence (**B**) and green fluorescence (**C**), respectively. Red and green fluorescent imagines were merged in (**D**). Note that when transformed with pmCherry-GFP, both mCherry and GFP were functional, leading to a merged yellow image.

To allow visual detection of mutations introduced by genome-editing enzymes, we made use of the fact that GFP and related proteins are often capable of retaining their function when present as a fusion protein. Thus, we generated a bacterial expression construct, pmCherry-GFP, that expresses an in-frame fusion protein of mCherry and GFP, with the coding regions of the fluorescent moieties separated by an in-frame multi-cloning linker, under the control of the Plac promoter (Fig. 1).

To test the function of the fusion protein, the fusion protein construct, an empty vector, or a vector expressing either GPP or mCherry was transformed into bacteria. After overnight plating, the bacterial colonies were visualized under bright field or fluorescent light. The results clearly showed that mCherry and GFP had their respective fluorescence when expressed either alone or as the fusion protein, while the colonies from the bacteria transformed with the empty vector had no fluorescence (Fig. 1).

### The loss of GFP fluorescence in pmCherry-GFP faithfully reports the presence of a frame shift in the multi-cloning linker region

**Figure 2:**
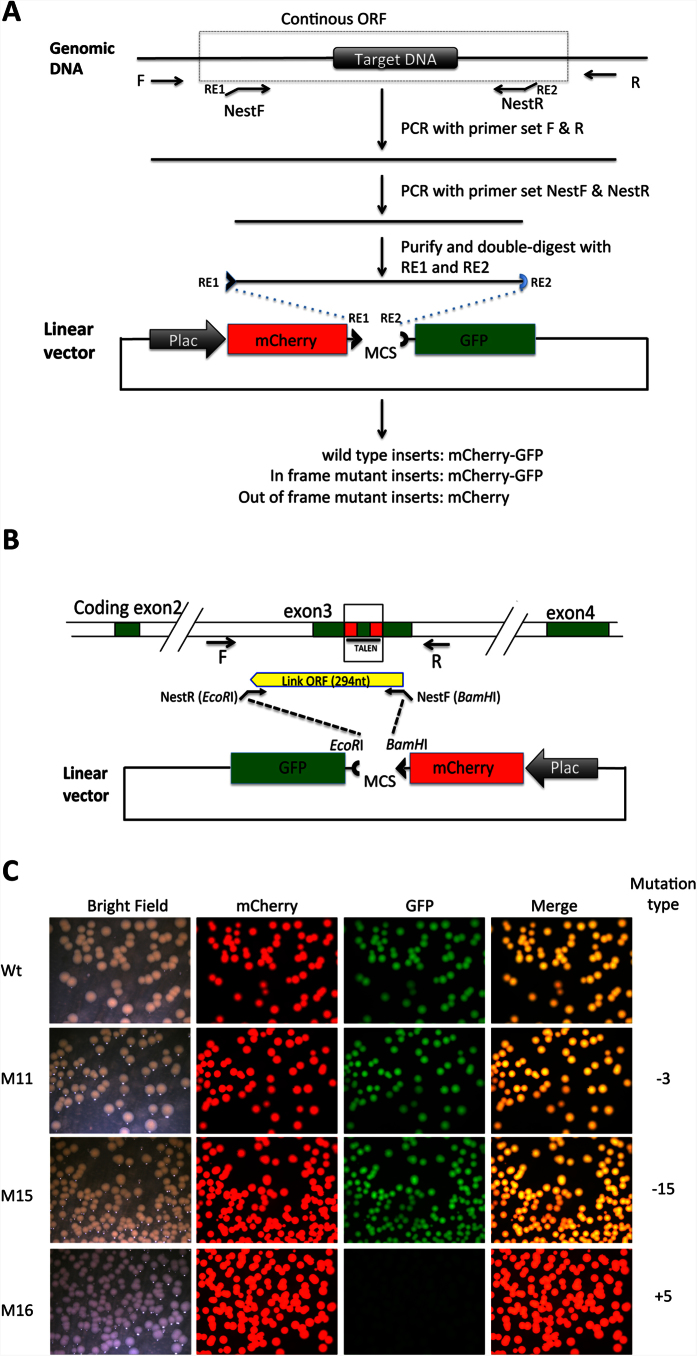
Shifting open reading frame upon inserting DNA into the MCS of the pmCherry-GFP construct leads to the loss of functional GFP moiety. (**A**) Diagram for cloning a genomic DNA fragment encompassing the region targeted by genome editing. Primers R and F are used to amplify a genomic fragment containing the targeted region. Two nested primers, NestF, with the recognition site by a restriction enzyme RE1 at the 5′-end, and NestR, with the recognition site by a restriction enzyme RE2 at the 5′-end, are designed to amplify a smaller fragment that is a continuous ORF in the wild type genome. The smaller fragment is purified and cleaved with RE1 and RE2 and dephosphorylated (to prevent self ligation of the fragment). The fragment is then cloned into pmCherry-GFP predigested with RE1 and RE2. The plasmid is then transformed into bacteria. If the PCR fragment is from wild type genomic DNA, a fusion protein of mCherry and GFP will be made in the bacteria. If genome editing leads to an in-frame mutation, a fusion protein of mCherry and GFP will also be made. If genome editing leads to an out-of-frame mutation or stop codon, the fusion protein will contain only mCherry but no functional GFP. (**B**). Diagram for cloning a genomic DNA fragment encompassing the region targeted by TALEN in the *Xenopus tropicalis* TRα gene. For the wild type genome, a fragment of 294 bp flanking the TALEN target site will be inserted into the pmCherry-GFP construct to produce a fusion protein with functional mCherry and GFP. (**C**). Inserting an out-of-frame but not an in-frame mutant fragment from the TRα gene disrupts GFP function. The PCR fragments amplified from the wild type DNA (Wt), two known in-frame deletion mutants (M11 and M15 with deletions of 3 and 15 bp, respectively) or an out-of-frame mutant (M16, with a 5 bp insertion) were cloned into pmCherry-GFP. The resulting plasmids were transformed into bacteria. The bacterial plates were visualized for mCherry and GFP. The merged mCherry and GFP images were shown in the last column.

To visualize and quantify mutations caused by genome-editing enzymes by using pmCherry-GFP, we designed primers to directionally clone the targeted region into the multi-cloning linker such that the unaffected, wild type genomic DNA fragment will maintain the frame to allow expression of a fusion protein of mCherry and GFP with a larger linker between the two moieties (Fig. 2A). Any mutation that introduces a stop codon or frame shift compared to the wild type fragment will therefore result in the expression of a truncated protein lacking GFP (Fig. 2A).

To test the validity of this approach, we used it to analyze a genomic DNA fragment encompassing the region targeted by a TALEN against *Xenopus tropicalis* thyroid hormone receptor (TR) α gene^16^. For the wild type genome, the PCR fragment was 294 bp flanking the TALEN target site, which when cloned into the pmCherry-GFP construct would produce a fusion protein of mCherry and GFP with 98 additional amino acids in the linker region between mCherry and GFP (Fig. 2B). As shown in Fig. 2C, the addition of the wild type fragment did not affect the expression and activity of either mCherry or GFP upon transformation of the resulting plasmid into bacteria. We next cloned a mutant DNA fragment with a 3 or 15 bp deletion or 5 bp insertion (M11, M15, M16, respectively) in the TALEN targeted region. When the resulting plasmids were transformed into bacteria, the two in-frame deletions, M11 and M 15, did not affect the expression or activity of either mCherry or GFP (Fig. 2C). In contrast, with a 5 bp insertion, the M16 fragment inactivated the GFP portion, leaving the bacterial transformants with only red fluorescence under fluorescent microscope (Fig. 2C). Thus, a mutation that causes a frame shift in the linker regions will disrupt the expression of the GFP moiety, leaving the bacterial colonies with only red fluorescence while in-frame mutations do not affect the expression and activity of either mCherry or GFP.

**Figure 3:**
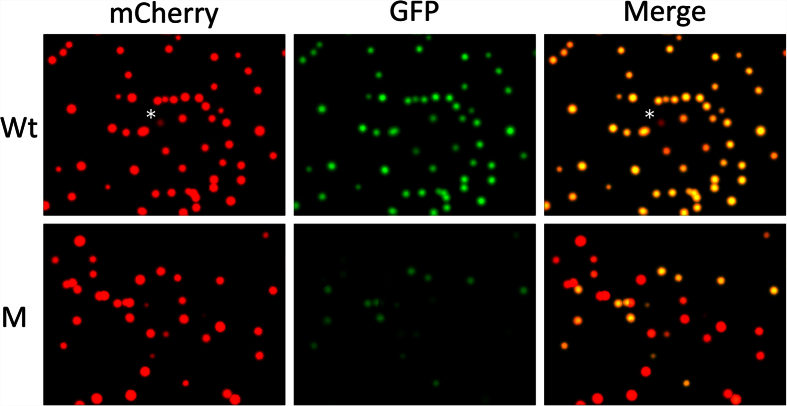
Quantifying out-of-frame mutation rate in F0 generation tadpoles produced with a TALEN targeting TRα gene in *Xenopus tropicalis* by using pmCherry-GFP construct. The mRNAs encoding the left and right arms of a TALEN targeting TRα as shown in Fig. 2B were injected into fertilized egg and the animals were reared into tadpole stage (see^16^ for details). A number of the resulting tadpoles (M) were euthanized together and the total genomic DNA was isolated. Genomic DNA was also isolated from wild type tadpoles (Wt). The TRα region from the Wt and M tadpoles was cloned into pmCherry-GFP and analyzed as shown in Fig. 2. Note that the yellow colonies in the merged panels represented the colonies with wild type TRα DNA inserted into pmCherry-GFP or colonies with in-frame mutations in the TRα DNA inserted into pmCherry-GFP. The red colonies in the merged image represented colonies with out-of-frame mutations in the TRα DNA inserted into pmCherry-GFP. *The single red colony from wild type genomic DNA plate. Sequencing of the colony revealed a point mutation leading to an in-frame stop codon in the TRα fragment, likely due to polymorphism at the site in the *Xenopus* genome or mutation introduced by PCR amplifications. Based on the number of red and yellow colonies in the merged panel, the out-of-frame mutation rate in the TRα gene due to TALEN can be determined (see Table 2 and text for details).

We next ask if the visualization assay can be used to quantify the out-of-frame mutation rate caused by the TALENs. The mRNAs encoding the left and right arms of the TALEN targeting TRα were injected into fertilized egg and the animals were reared into tadpole stage (see^16^ for details). A randomly selected group of the resulting tadpoles were euthanized together and the total genomic DNA was isolated. For comparison, genomic DNA was also isolated from wild type tadpoles. The TALEN-targeted region in the TRα gene was PCR-amplified and cloned into pmCherry-GFP. The plasmid was then transformed into bacteria. As shown in Fig. 3, inserting the TRα DNA from wild type tadpoles into the pmCherry-GFP produced colonies that had both mCherry and GFP fluorescence, consistent with the in-frame nature of the insertion of the wild type fragment. In contrast, when the DNA from TALEN-injected tadpoles was cloned into pmCherry-GFP, the resulting transformed bacterial colonies were a mixture of GFP+ and GFP−ones (Fig. 3). Quantitative analysis showed that the GFP− colonies (essentially all out-of-frame deletion/insertion mutants since the possibility of in-frame stop codon mutations is very low) represented 46.9 ± 1.7% from TALEN-treated animals and 0.5 ± 0.5% of the total colonies from wild type animals (Table 2) (Note that sequencing of such colonies derived from wild type genomic DNA for TRα as well as Sox3 described below found out-of-frame deletions in the PCR primers, likely due to errors in primer synthesis, or in-frame stop codons outside of the TALEN target regions in the genomic DNA, likely due to genomic DNA polymorphism or PCR-amplification errors (data not shown)). To validate if the out-of frame mutation rate determined from the color assay reflected the actual out-of-frame mutation rate of the genomic DNA from TALEN-treated animals, we sequenced all colonies from one of LB agar plates. Sequence analysis revealed that 46.3% (19 out of 41 colonies) had out-of-frame mutations (Supplemental Fig. 4), consistent with color assay.

**Figure 4:**
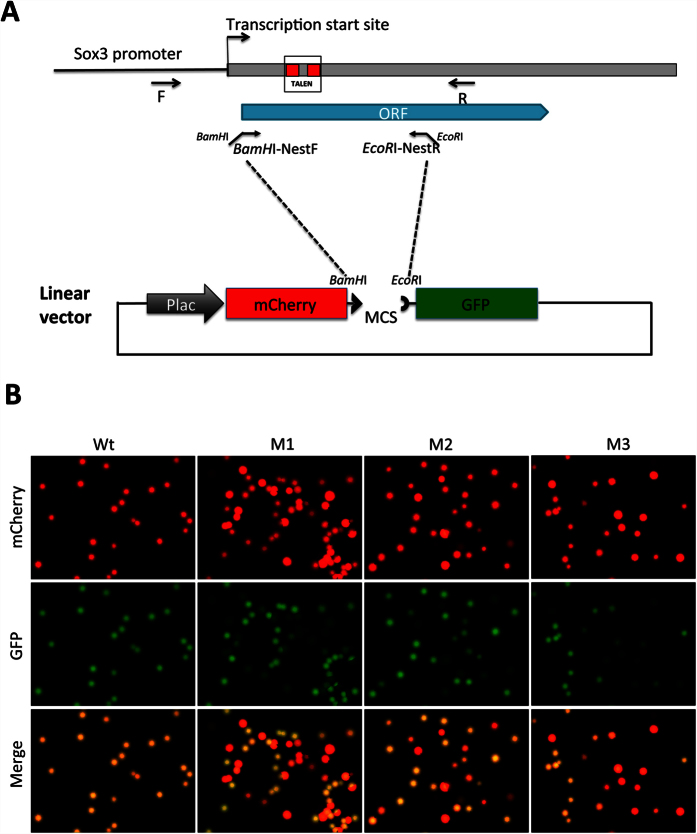
Analyzing mutations in F0 generation tadpoles produced with a TALEN targeting Sox3 gene in *Xenopus tropicalis* by using pmCherry-GFP construct. (**A**) Diagram showing the strategy to clone a genomic DNA fragment encompassing the region targeted by TALEN in the *Xenopus tropicalis* Sox3 gene into pmCherry-GFP construct. (**B**) Three different tadpoles generated from eggs injected with Sox3 TALEN have different rate of mutations in the Sox3 gene. The mRNAs encoding the left and right arms of a TALEN targeting the Sox3 region shown in (**A**) were injected into fertilized egg and the animals were reared into tadpole stage. The genomic DNA was isolated from one wild type (Wt) and 3 different F0 Sox3 TALEN tadpoles (M1-3), cloned into pmCherry-GFP construct and analyzed as shown in Figs 2 and 3. Note that all colonies from the Wt sample were yellow in the merged image while for the mutant samples, some were red and some were yellow.

To ensure the general utilization of our approach, we next used it to analyze the mutations caused by a novel TALEN against the *Xenopus tropicalis* Sox3 gene, a TR target gene during adult stem cell development^17^ (Fig. 4). The mRNAs encoding the left and right arms of the TALEN targeting Sox3 (Fig. 4A) were injected into fertilized egg and the animals were reared into tadpole stage. One wild type and 3 Sox3 TALEN-injected tadpoles were randomly chosen for genomic DNA isolation. The TALEN targeted region in the Sox3 gene was PCR amplified and cloned into pmCherry-GFP. The resulting plasmids were transformed into bacteria and analyzed as before. As shown in Fig. 4, essentially all colonies from the wild type tadpole sample had both red and green fluorescence and appeared as yellow in the merged image of the red and green fluorescence. The colonies from the mutant samples were a mixture of red and yellow in the merged image, indicating some had out-of-frame mutations in the Sox fragment (thus, lacking GFP fluorescence). Sequencing of some randomly selected GFP− and + colonies in the TALEN-treated tadpoles confirmed that all GFP- colonies had out-of-frame mutations while all GFP+ colonies had wild type or in-frame mutations in the TALEN targeted region of Sox3 gene (Supplemental Fig. 1). To determine if the GFP color assay could quantify the out-of-frame mutation rate, we analyzed the DNA from one mutant animal in detail. GFP color assay revealed that 41.2 ± 1.3% of the colonies were out-of-frame mutant (see Sox3 in Table 2). Again, GFP − colonies from the wild type control tadpole (the background) were 1.1 ± 0.4%. Importantly, when we sequenced all colonies from one of plates for the mutant DNA, we observed that 42.9% (18 out of 42) had out-of-frame mutations (Supplemental Fig. 4), again in agreement with the direct color assay.

### The fluorescent moieties in the pmCherry-GFP construct can be replaced with other reporter groups

**Figure 5:**
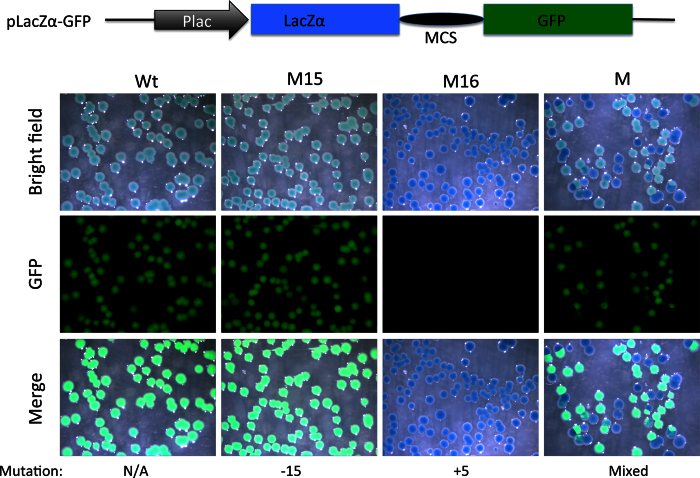
Substitution of mCherry coding region with that of LacZα allows detection of out-of-frame mutations with a combination of X-gal staining and a simple fluorescent microscope (without a need to resolve red vs. green fluorescence). A PCR fragment flanking the TALEN targeting site in TRα gene in Xenopus tropicalis was amplified with the NestF(*BamH*I) and NEstR(*EcoR*I) from genomic DNA isolated from a wild type tadpole (Wt), a tadpole with a known in-frame deletion (M15 with a deletion of 15 bp) or an out-of-frame mutant (M16, with a 5 bp insertion) shown in Fig. 2C, or mutant F0 TRα TALEN tadpoles (Fig. 3). The amplified fragment was cloned into the *BamH*I and *EcoR*I double-digested pLacZα-GFP (same as pmCherry-GFP except the mCherry sequence has been replaced with LacZα sequence, illustrated on top). The resulting plasmids were transformed into bacteria. The bacterial plates were stained with X-gal. Pictures were taken under the bright field (Bright Field) to visualize X-gal staining (blue) or under a fluorescent microscope for GFP. Note that colonies from plasmid containing Wt DNA or DNA with the in-frame deletion of 15 bp were greenish blue under the bright field, likely reflecting the blue color of the X-gal staining plus weak green color due to the GFP under visible light. On the other hand, a 5 bp insertion caused frame shift and disrupted GFP function, leading to pure blue colonies in the bright field or merged images. Colonies from the mutant tadpole (M) were a mixture of blue (lack of GFP) or greenish blue colonies in the bright field.

Our approach relies on the expression of a fusion protein with two different functional moieties for color visualization. In theory, both the mCherry and GFP moieties can be replaced with other reporter groups to expand the potential range of the applications. To test this possibility, we replaced the mCherry coding region in the pmCherry-GFP construct with the coding region for LacZα. Transformation of the resulting plasmid, pLacZα-GFP, would led to the expression of the fusion protein LacZα-GFP, which could be detected by X-gal staining for visualization of LacZα under bright field (blue) or with a fluorescent microscope for GFP (thus there is no need to distinguish different fluorescent colors). To determine if this construct can be used to detect out-of-frame mutations, the PCR fragment flanking the TALEN targeting site in TRα gene (see above) was amplified from a wild type tadpole, a mutant DNA with a known in-frame deletion (M15 with a deletion of 15 bp) or an out-of-frame insertion (M16, with a 5 bp insertion, see Fig. 2C), or TRα TALEN-injected tadpoles (Fig. 3), and cloned into the pLacZα-GFP. The resulting plasmids were transformed into bacteria. The bacterial plates were stained with X-gal (blue) or photographed under a fluorescent microscope for GFP. As shown in Fig. 5, all colonies from plasmid containing wild type DNA or DNA with the in-frame deletion of 15 bp were greenish blue under the bright field, likely reflecting a slightly weaker blue color of the X-gal staining, which was likely due to the in-frame GFP fusion to LacZα, plus weak green color by the GFP moiety under visible light. They were also GFP+ under a fluorescent microscope. On the other hand, the colonies from the mutant DNA with a 5 bp insertion were pure blue in the bright field or merged images, lacking any GFP fluorescence. The colonies from the mutant tadpoles injected with the TALEN were a mixture of blue (lack of GFP) or greenish blue colonies. Thus, the mCherry group can be replaced with LacZα. Interestingly, giving the visible fluorescence of GFP even under bright field, we could easily identify the out-of-frame mutations with just bright field visualization of the colonies, making the approach useful even when a fluorescent microscope is not available.

To test the application of this approach for other targeted regions in the genome, we used it to analyze mutations at four other loci, i.e., Sox3, TRα-LBD (ligand binding domain), which is a region different from the TRα target analyzed in Figs 3 and 5, and two TALEN targeted regions in Dot1L (disruptor of telomeric silencing (Dot) 1-like) in *Xenopus tropicalis* genome^14^. Again, we observed that the colonies from wild type genomic DNA loci were greenish blue while those from animals injected with TALEN-mRNAs were mixture of greenish blue and blue colonies under bright field microscope (Supplemental Fig. 2). Consistently, the greenish blue colonies had bright GFP signal under a fluorescent microscope, independent of the genomic loci being analyzed (Supplemental Fig. 2). We chose 15 random colonies that were blue under a bright field microscope and GFP-negative under a fluorescent microscope for each of the four loci in quest to perform analysis. Except 3 colonies for a Dot1L TALEN1 target region that lacked the insert but had deletions around the BamHI and EcoRI sites, all the other 57 colonies contained the respective DNA targets and had out-of-frame mutations within the target regions (Supplemental Fig. 3), confirming the prediction based on the color detection. In addition, the out-of-mutation rates as determined based on the pLacZα-GFP color assay for TRα-LBD and the two TALEN targeted regions in Dot1L were all in agreement with the actual out-of-frame mutation rates determined by sequencing all colonies of a plate of about 40–50 colonies for each targeted region (Table 2 and Supplemental Fig. 4).

## Conclusion

Rapid and easy identification of mutants is crucial for the ever-expanding applications of genome-editing tools in diverse research and biological/biomedical product development. While many detection methods have been developed, our approach here offers several distinct advantages. First, it is a simple visual protocol that can be carried out even without a fluorescent microscope (with pLacZα-GFP) or a simple fluorescent microscope (with pLacZα-GFP or pmCherry-GFP). Second, it is quantitative, allowing easy determination of mutational efficiency of the genome-editing enzymes, making it very useful for screening good genome-editing enzymes. Finally, compared to other approaches, it is the only reliable one, other than direct sequencing, that allows easy identification of desired mutants, i.e., those that inactivate the targeted genes by introducing either in-frame stop codons or out-of-frame insertions/deletions.

Our approach is built upon the fact that protein moieties are capable of functioning independently even when present in a large fusion protein. Thus, we introduce a linker with multiple cloning sites to separate the in-frame coding regions for two reporter moieties: mCherry and GFP in pmCherry-GFP or LacZα and GFP in pLacZα-GFP, respectively. This allows us to clone any DNA fragments of interest into the linker region. Any DNA fragments with in-frame stop codons or out-of-frame insertions/deletions will disable the expression of the second reporter, the GFP moiety and thus can be visually and quantitatively detected upon transformation of the resulting plasmid into bacteria.

A previous report had used a single color reporter, i.e., LacZ recovery/disruption assay, to quantify mutation rate from genome editing^13^. When we cloned the wild type and mutant DNA used in Fig. 2C into such a reporter (pUC19 vector, parent vector for making the pmCherry-GFP and pLacZα-GFP), we observed, as expected, that wild type DNA and in-frame mutant DNA led to blue colonies. Surprisingly, colonies from the out-of-frame mutant (M16, see Fig. 2C) were blue (although a little weaker), instead of being white (data not shown), likely due to the use of alternative translational start site(s). This makes it necessary to carefully judge the signal intensity in order to identify the out-of-frame mutants when using a single color LacZ methods, contrasting sharply with the unmistakable difference observed with our approaches (Figs 2 and 5). In addition, we also noticed that white satellite colonies from the wild type or in-frame mutant samples, giving rise to false positive counts of out-of-frame mutants, especially when the colonies were at high density (data not shown). In contrast, our approach use two in-frame reporter genes: the first reporter, i.e. mCherry or LacZα, serves as an internal control against contaminated or satellite white colonies, while the second one allows the detection of the out-of-frame mutations, avoiding any potential confusion.

We have applied our approach to analyze F0 generation *Xenopus tropicalis* tadpoles generated by using TALENs against several different loci in different genes, such as the thyroid hormone receptor TRα and transcription factor Sox3. In all cases, our method revealed the presence of out-of-frame deletions/insertions in the F0 tadpoles, much simpler and easier than sequencing that is typically required. Furthermore, sequencing individual bacterial colonies confirmed the presence of out-of-frame deletions/insertions as our color assay predicted and the out-of-mutation rates obtained from the color assays on all 5 different TALEN targets matched the out-of-mutation rates determined from direct sequencing analysis (Table 2 and Supplemental Fig. 4). This makes it possible to use our color detection for accurate quantification of the rate of out-of-frame mutations (Table 2).

Our visual detection approach also greatly simplified the requirement for identification of mutants and screening for good genome-editing enzymes as it by-passes the need for sequencing and allows the identification of the desired, gene-disrupting mutants that other non-sequencing methods cannot. Furthermore, by replacing mCherry with LacZα, we have shown that we can even use simple cloning and bright field visualization to identify and quantify mutations. This latter approach even eliminates the needs for a fluorescent microscope, often an expensive piece of equipment that is not available to many research groups. Thus, our approach should vastly expand the application of genome-editing to diverse research groups and fields.

## Additional Information

**How to cite this article**: Fu, L. *et al*. A simple and efficient method to visualize and quantify the efficiency of chromosomal mutations from genome editing. *Sci. Rep.* **6**, 35488; doi: 10.1038/srep35488 (2016).

## Supplementary Material

Supplementary Information

## Figures and Tables

**Table 1:** Primers for PCR-amplification of the TALEN target regions.

Gene	Primer	Sequences	Note
TR**α**	F	5′-ATTGGGTTGGTTGTGGGTCG-3′	
	R	5′-TAAGGTGGGGGCAGTACAGG-3′	
	NestF	5′-CCCTCG**GGATCC**ATACTGCGGTAGTGG-3′	BamHI in bold
	NestR	5′-CAGTTT**GAATTC**TTTTGATGACTGTTTTG-3′	EcoRI in bold
Sox3	F	5′-GCTTTAGCGCGCATCACACCTG-3′	
	R	5′-CTGATACTTGCCAGGCAAGCAAAGC-3'	
	NestF	5′-GCAG**GGATCC**GGGAAGTTTGTGCCGGGATC-3′	BamHI in bold
	NestR	5′-CCAGTT**GAATTC**CCGCTCCTGATCCGGGATAG-3′	EcoRI in bold

**Table 2:** Quantitative analysis of out-of-frame mutations caused by TALENs.

Gene		Color assay Mean % ± S.E	Sequence analysis % (Out-of-frame/Total colonies)
TRα-DBD	Wild type	0.5 ± 0.5%	
	Mutant	46.9 ± 1.7%	46.3% (19/41)
Sox3	Wild type	1.1 ± 0.4%	
	Mutant	41.2 ± 1.3%	42.9% (18/42)
TRα-LBD	Wild type	4.4 ± 0.3%	
	Mutant	26.1 ± 0.6%	25.0% (12/48)
Dot1L-TALEN1	Wild type	3.2 ± 0.2%	
	Mutant	37.2 ± 0.8%	37.8% (17/45)
Dot1L-TALEN2	Wild type	2.9 ± 0.5%	
	Mutant	46.3 ± 1.4%	45.2% (19/42)
