# Supplementary material for: A simple and efficient method to visualize and quantify the efficiency of chromosomal mutations from genome editing
Source: Sci Rep. 2016 Oct 17;6:35488. doi: 10.1038/srep35488 (PMC5066342; doi:10.1038/srep35488)
Supplement: Supplementary Information [file srep35488-s1.pdf]

A simple and efficient method to visualize and quantify the efficiency of chromosomal mutations from genome editing.

Liezhen Fu, Luan Wen, Nga Luu and Yun-Bo Shi

Section on Molecular Morphogenesis, Eunice Kennedy Shriver National Institute of Child Health and Human Development (NICHD), National Institutes of Health (NIH), 18 Library Dr., Bethesda, Maryland, 20892

Corresponding Authors:

Yun-Bo Shi, (301) 402-1004, (301) 402-1323 (fax), Shi@helix.nih.gov

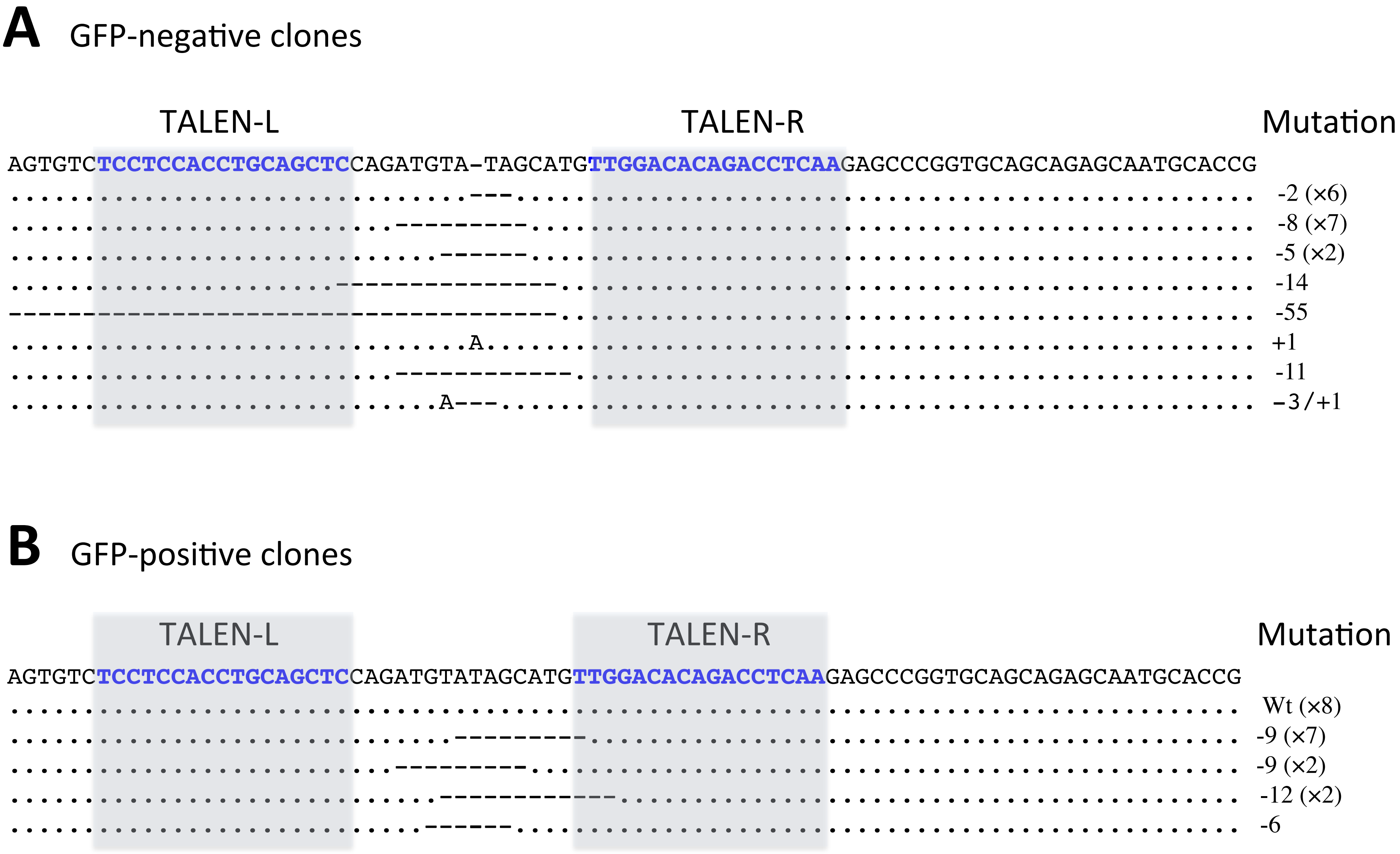

Supplemental Fig. 1

Supplemental figure 1: Sequencing of GFP+ and GFP- colonies confirms that a positive GFP signal is indicative of a wild type or in-frame mutation while the absence of GFP signal indicates an out-of-frame mutation at the TALEN-targeted locus of the genome in animals injected with TALEN mRNAs targeting Xenoups tropicalis Sox3 gene.

The mRNAs encoding the left and right arms of a TALEN targeting the Sox3 region shown in Figure 4A were injected into fertilized eggs and the animals were reared into tadpole stages. The genomic DNA was isolated from the tadpoles, PCR amplified, and cloned into pmCherry-GFP construct and analyzed as shown in Figs. 2 and 4. Colonies with red fluorescence of mCherry expression were selected and sorted into GFP-negative (A) and GFP-positive (B) groups, grew in LB medium supplemented with ampicillin. Plasmid DNA was isolated from them and subjected to DNA sequence analysis. TALEN targeted sequences are in blue and the areas are shaded. Note that all the GFP-negative clones contained out-of-frame mutations while the GFP-positive clones were either wild type or in-frame mutants. The mutations with deletions (-) and additions (+) were indicated. When there were more than one colony of a particular mutation, the number of colonies was shown in parenthesis.

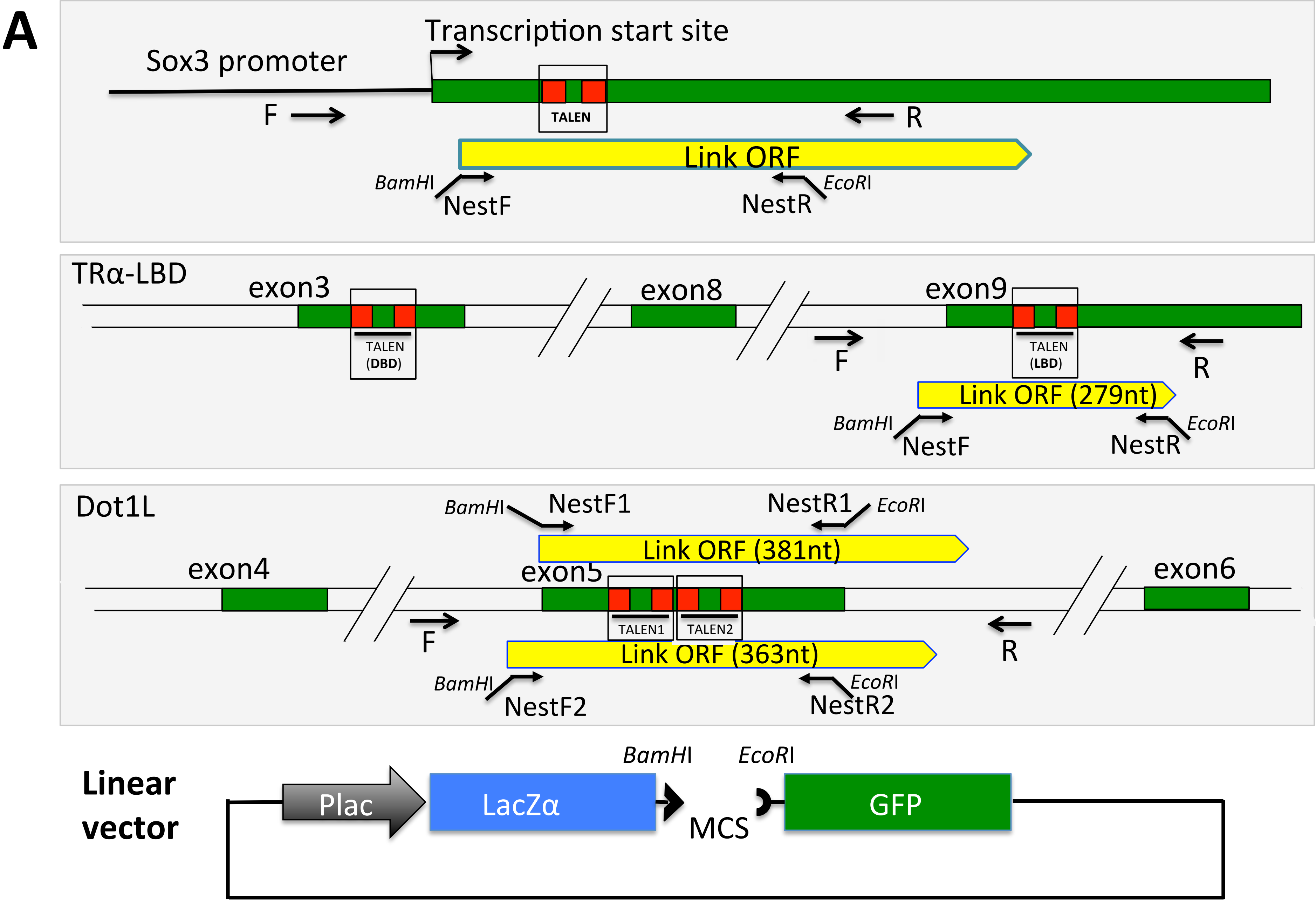

Supplemental Fig. 2

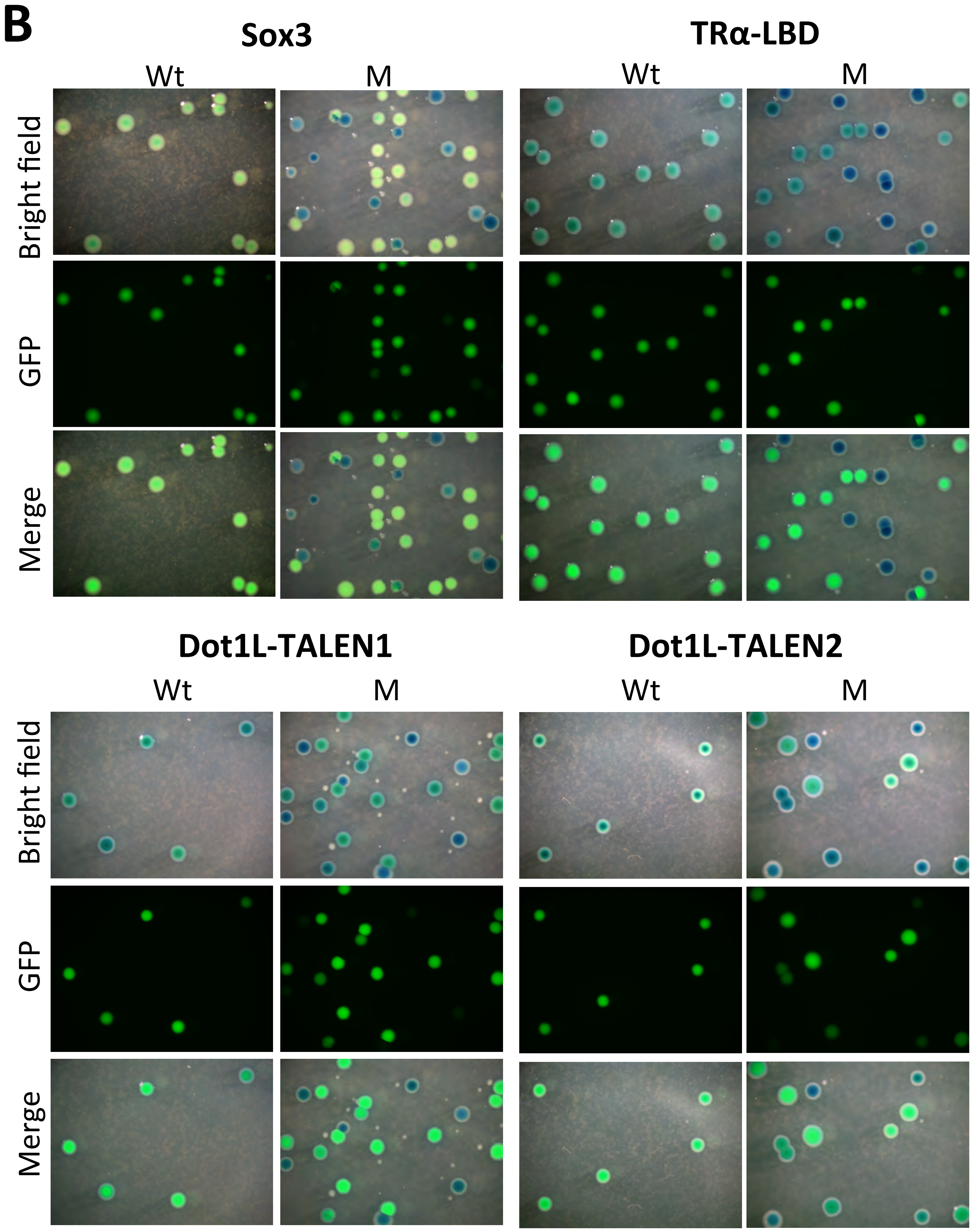

Supplemental Fig. 2

Supplemental figure 2: Detection of out-of-frame mutations induced by four other TALENs with a combination of X-gal staining and GFP fluorescence.

A) Diagram showing the strategy to clone a genomic DNA fragment encompassing the regions targeted by TALENs in *Xenopus tropicalis*. A TALEN for *Xenopus tropicalis* Sox3 (top) were custom-designed and assembled by Cellectis Bioresearch, Inc. (Cambridge, MA, USA) to target the region around the start codon of the gene as described in Material and Methods. The TALENs targeting *Xenopus tropicalis* TRα ligand binding domain (TRα-LBD), Dot1L (TALEN1 and TALEN2), respectively, were custom designed and assembled as previously described (Wen L and Shi Y-B, 2015, Endocrinology, 156(2):721–734; Wen et al., 2015, FASEB J, 29, 385– 393). The TRα-LBD left (TRα-LBD-L) arm recognizes the sequence TCCCCACTTCTGGCCC and the TRα-LBD right (TRα-LBD-L) arm recognizes the sequence TCATGCGCAGGTCCGTCACC in the TRα-LBD region. The Dot1L TALEN1 left (TALEN1-L) arm recognizes the sequence GAAAAACTCAACAA and the TALEN1 right (TALEN1-R) arm recognizes the sequence TCTCCATAGACCTCA in the Dot1L coding region. The Dot1L TALEN2 left (TALEN2-L) arm recognizes the sequence TACTGGTCTCCTTCGC and the TALEN2 right (TALEN2-R) arm recognizes the sequence TGTTACAGAGTGGTTGTAGAC in the Dot1L coding region. The TALEN mRNAs were synthesized in vitro and injected into fertilized eggs as described in the Materials and Methods. A pair of PCR primers (F and R) and a pair of nested PCR primers (NestedF, with an added BamHI recognition site at its 5'-end; and NestedR, with an added EcoR1 recognition site at its 5'-end) for each target region were designed for PCR amplification (see supplemental Table1) and subsequent cloning of the nested PCR fragment into pLacZα-GFP construct as described in the Materials and Methods. Note that the Dot1L TALEN1 and TALEN2 target adjacent region and share the F and R primers. The TRα-LBD TALENs (TALEN (LBD)) target the exon9, a region far away from the TRα TALENs targeting the DNA binding domain shown (TALEN(DBD) targets the exon3) which was analyzed in Fig.2, 3 and 5.

B) PCR fragments flanking the TALEN targeting sites in Sox3, TRα-LBD, or Dot1L (Dot1L-TALEN1 and Dot1L-TALEN2) genes were amplified with the NestF(*BamH*I) and NestR(*EcoR*I) from genomic DNA isolated from a wild type tadpole (Wt) and F0 TALEN-injected tadpoles (M). The amplified fragments were double-digested with BamHI and EcoRI followed by calf intesitinal alkaline phosphatase treatment, and cloned into the *BamH*I and *EcoR*I double-digested pLacZα-GFP as described in Fig.5. The resulting plasmids were transformed into bacteria. The bacterial plates were stained with X-gal. Pictures were taken under the bright field (Bright Field) to visualize X-gal staining (blue) or under a fluorescent microscope for GFP. Note that the colonies from plasmid containing Wt DNA at different loci were uniformly greenish under the bright field, likely reflecting the blue color of the X-gal staining plus weak green color due to the GFP under visible light. On the other hand, colonies from the mutant tadpole (M) were a mixture of blue (lack of GFP) or greenish colonies in the bright field. The greenish colonies for both Wt and mutant animals had strong GFP signal under a fluorescent microscope for GFP. Three independent samples from TALENs-injected tadpoles were analyzed for each TALEN target and a representative picture is shown in the figure. The experiments were repeated twice.

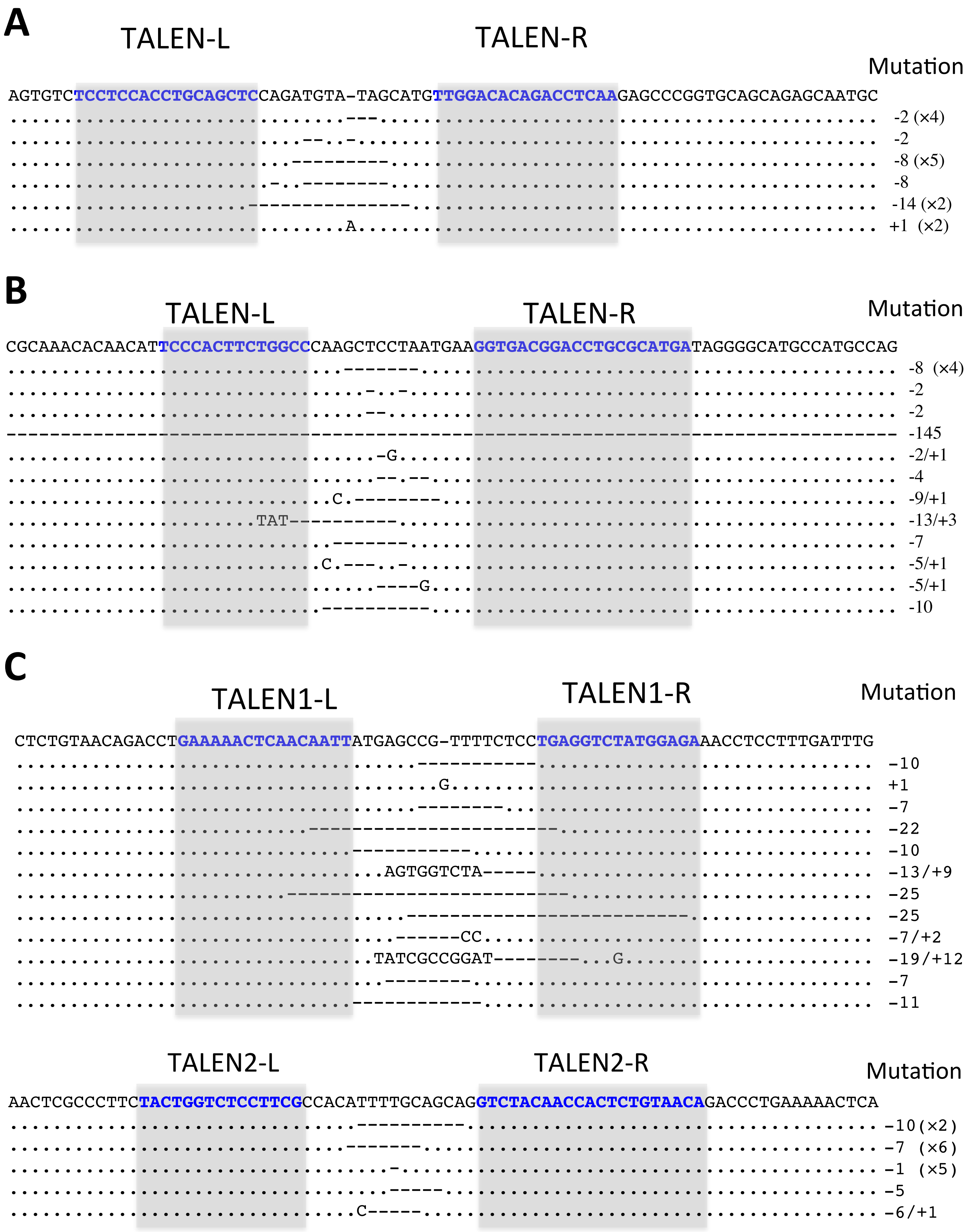

Supplemental Fig. 3

Supplemental figure 3: Sequence analysis confirms that GFP-negative clones were out-of-frame mutants.

The mRNAs encoding the left and right arms of a TALEN targeting the Sox3 (A), TRα-LBD (B) or Dot1L (C, TALEN1 and TALEN2, respectively) region shown in Supplemental Fig. 2A were injected into fertilized eggs and the animals were reared into tadpole stage. The genomic DNA was isolated from the tadpoles, PCR amplified, cloned into pLacZα-GFP construct and analyzed as shown in Supplemental Fig. 2B. Blue colonies under a bright field microscope and GFP-negative under a fluorescent microscope were randomly picked up and grew in LB medium supplemented with ampicillin. Plasmid DNA was isolated from them and subjected to DNA sequence analysis. TALEN targeted sequences are in blue and the areas are shaded. Note that all the clones had out-of-frame mutations as predicted by the lack of GFP signal.

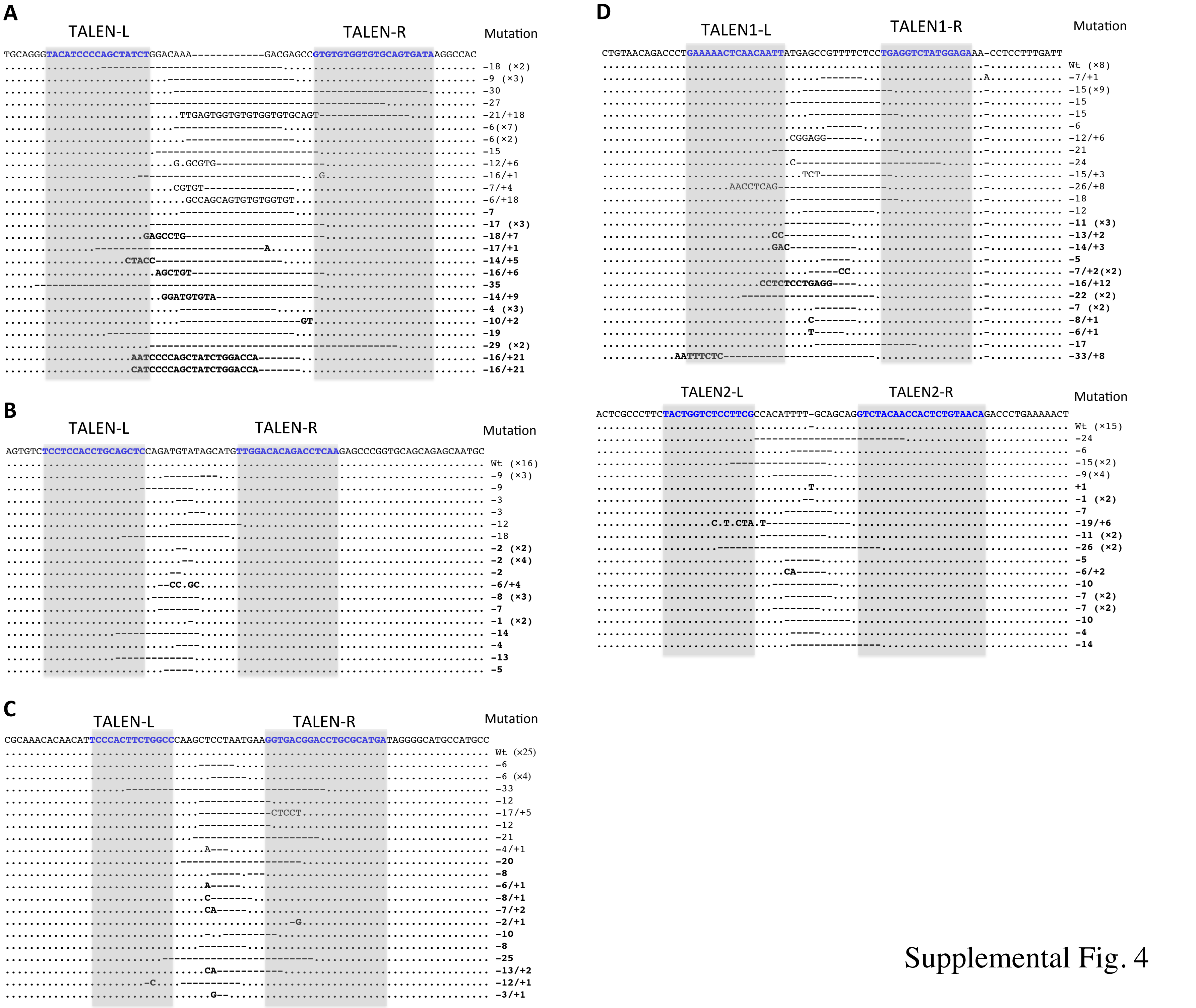

Supplemental Fig. 4

Supplemental figure 4: Sequence analysis confirms that out-of-mutation rates determined from the two-color assay reflects the actual out-of-mutation rates within the TALEN targeted regions.

The mRNAs encoding the left and right arms of a TALEN targeting the TRα-DBD (A), Sox3 (B), TRα-LBD (C) or Dot1L (D, TALEN1 and TALEN2, respectively) region shown in Fig. 2B, Fig. 3A and Supplemental Fig. 2A were injected into fertilized eggs and the animals were reared into tadpole stages. The genomic DNA was isolated from the tadpoles, PCR amplified, cloned into pmCherry-GFP (for TRα-DBD and Sox3) or pLacZα-GFP (for TRα-LBD and Dot1L TALEN1 and TALEN2, respectively) constructs and analyzed as indicated in Material and Methods. All colonies on a plate containing about 40 to 50 colonies for each target were picked up and grew in LB medium supplemented with ampicillin. The plasmid DNA was isolated and subjected to DNA sequencing. The TALEN targeted sequences are in blue and the areas are shaded. The mutations with deletions (-) and additions (+) were indicated. When there was more than one colony of a particular mutation, the number of colonies was shown in the parenthesis. Out-of-frame mutations were in bold letters. The out-of-frame mutation rates were calculated by dividing the numbers of out-of-frame mutants by the total clones containing the respective TALEN target sequences and summarized in Table 2.

Supplemental Table 1: Primers for PCR-amplification of the TALEN target regions shown in Supplemental Fig. 2

Gene	Primer	Sequences	Note
TRα-LBD	F	5’-GATGCCATCTTTGACCTTGG -3’	
	R	5’-ACTTCCTGGTCCTCAAAGAC-3’	
	NestF	5’-GACTCTAGA**GGATCC**AGACCGTACTGGTTTAATGTGC -3’	BamHI in bold
	NestR	5’-CCAGTT**GAATTC**ACCTCAAGGAAGAGCGGTGGAA -3’	EcoRI in bold
Dot1L	F	5’-GAAAGCCACATTGAAGTGGCAC-3’	
	R	5’-TGCAAAAAGGAGCACCAGCCTG-3'	
	NestF1	5’-GACTCTAGA**GGATCC**GTGGAAAGGCACCACTCATCC -3’	BamHI in bold
	NestR1	5’-CCAGTT**GAATTC**CACTTCCCAGGTCCACAAAG -3’	EcoRI in bold
	NestF2	5’-GACTCTAGA**GGATCC**CATGACAACTACATATTGCATC -3’	BamHI in bold
	NestR2	5’-CCAGTT**GAATTC**AGGTCCACAAAGAGATCATCC -3’	EcoRI in bold
